# Netiquette: Ethic, Education, and Behavior on Internet—A Systematic Literature Review

**DOI:** 10.3390/ijerph18031212

**Published:** 2021-01-29

**Authors:** Rebeca Soler-Costa, Pablo Lafarga-Ostáriz, Marta Mauri-Medrano, Antonio-José Moreno-Guerrero

**Affiliations:** 1 Department of Education Sciences, University of Zaragoza, 50009 Zaragoza, Spain; rsoler@unizar.es (R.S.-C.); mmauri@unizar.es (M.M.-M.); 2 Department of Didactics and School Organization, University of Granada, 51001 Ceuta, Spain; ajmoreno@ugr.es

**Keywords:** netiquette, systematic review, social media, digital competence, ICT

## Abstract

In this article, an analysis of the existing literature is carried out. It focused on the netiquette (country, date, objectives, methodological design, main variables, sample details, and measurement methods) included in the Web of Science and Scopus databases. This systematic review of the literature has been developed entirely according to the Preferred Reporting Items for Systematic Reviews (PRISMA). The initial search yielded 53 results, of which 18 exceeded the inclusion criteria and were analyzed in detail. These results show that this is a poorly defined line of research, both in theory and in practice. There is a need to update the theoretical framework and an analysis of the empirical proposals, whose samples are supported by students or similar. Knowing, understanding, and analyzing netiquette is a necessity in a society in which information and communication technologies (ICT) have changed the way of socializing and communicating. A new reality in which there is cyber-bullying, digital scams, fake news, and haters on social networks.

## 1. Introduction

Billions of people have taken an active part in technological development over the past decade. Social networks have been the maximum exponent of a digital revolution that has meant a before and after in terms of how people communicate and collaborate [1]. A new reality that has been mutating from the original Facebook, YouTube, and Flickr [2] to become, for example, a relevant strategy in election campaigns [3,4,5]. The perfect framework for its expansion has been mobile devices, especially smartphones [6,7]. Both its technical conditions and its rapid incorporation into almost any area of life today [8] have made it the perfect nexus. In fact, nowadays it is not surprising that applications such as Instagram [9], or others more associated with instant messaging [10], are among the most frequented practices when accessing these mobile devices [11].

The popularity and growth of social networks can be understood by the paradigm shift that brought about their birth, as previously the World Wide Web was based on limited usability. Its appearance meant that users were already able to create, modify, share, and discuss existing content on the Internet [12]. Consequently, the attractiveness of using these digital media was no longer governed only by the content, but also by the new possibilities of participation they offered [13]. Although it has not been total, as the digital divide is still present [14,15,16,17], one of the technological consequences of the second decade of the 21st century has been the easy access to these new opportunities [18]. Despite cultural differences and resistance [19], it is clear that the option of being part of and participating in these digital communities [20] has been reduced to a couple of keystrokes.

This new digital map, which over the years has unlocked new horizons, has altered such basic habits as what to take with you when you leave home: wallet, keys, and smartphone [21]. This is an everyday action that can be understood in the face of the digital approach to modern life [22] and which perfectly contextualizes the rise of digital marketing [23]. Scientific production in this area has developed considerably [24,25] in response to the need to understand, know, and adapt commerce in view of the new forms of relationship and consumption that have emerged as a result of social networks [26,27]. A new ecosystem implies an almost total rethinking of roles [28], as reflected in the so-called influencers [29], and of strategies in an environment that is struggling to obtain the greatest dissemination and impact, including for health reasons [30].

This is an interest that responds to a historical moment in which social networks offer the right possibilities to cover everything from the individual to the social [31], including work [32]. Some of the main characteristics that may justify this phenomenon are: being a quick and easy alternative to access a wide range of information [33,34], offering almost instantaneous interaction and communication [35], opening up business possibilities in global environments and not just local ones [36], and even serving as a parallel strategy to find out preferences and interests in particular issues [37]. These applications accompany the new digital habits, especially among the young, whose interest in the more classic media is declining and who are developing new digital skills in areas such as content consumption [38].

The so-called digital competence (DC) is the theoretical approach to this new panorama specifically in the educational field [39]. A concept that corroborates the impact of digital technology on personal development [40], which means that educational institutions must rethink their approach in light of the new needs and demands which this generates [41]. Since its incorporation in 2006, scientific interest has been increasing and evolving. Approaches have sought to explore key issues such as what the DC is in a changing technological context [42], particularly in light of legislative frameworks [43]. The most evaluative orientation has been toward understanding the level of DC in different educational contexts: educational stages [44,45] or agents [46]. Perspectives that respond to a context where the integration of ICT in schools occurs from the technical [47] to the methodological [48], especially during the Covid-19 pandemic [49].

Digital media can contribute, as in the educational field [50], but their use can also lead to harm. Recently, problems have emerged such as addiction to smartphones, known as nomophobia [51], whose relationship with anxiety and stress [52] shows that ICTs also have a negative side. Cyber-bullying [53,54] is another example of how the use of technology can be negative, similar to the anonymity of social networks as a weapon of hate [55]. Information, one of the main reasons for the use of networks, is also under scrutiny following the rise of fake news [56] and the use of user data is also controversial [57]. At the same time, consumer advocacy is growing [58], reflected in the presence of cookies on any website and “integrated shopping” in free downloadable applications.

This new framework, with its possibilities and repercussions [59], gives rise to another approach more related to how they have used: netiquette. The origin of this term, which is based on the link between the words Internet and label [60,61], emerges on the eve of the beginning of the 21st century and the expansion of the digital world. The Internet, as well as promoting access to knowledge or creating new professions, has created the non-face-to-face label. This can be seen in traditional face-to-face customs such as giving condolences, the development of which through social networks, especially Facebook, has become standardized [62]. A revolution that entails extrapolating civic norms from the face-to-face to the digital in a technologically interconnected world [63]. Guidelines, which are less or more assimilated, are present in couple relationships [64] or which guide the use of such essential tools as email [65,66] in work environments [67,68,69,70].

To talk about ethics or a social label is really to talk about education. It is therefore not surprising that in a context of constant inclusion of ICT in the classroom [71], netiquette is one of the areas that make up the DC. A key training requirement in current and future teachers whose preparation in the digital field continues to be analyzed [72,73], more so when it is a field in evolution since, above all, social networks are altering and promoting new digital habits in students [74,75]. In the case of the educational field, the pandemic has highlighted the role of ICTs [76], a reality which means understanding the digital label as part of human development in the 21st century. An approach that has been focused above all on students who have grown up with the digital [77] but which, in reality, is already inherent to anyone who has access to a mobile device with an Internet connection.

The present study is based on this new paradigm. Access to digital media is already a routine, even an addiction, and it is urgent to understand its new social patterns. In the educational field, especially in training, this idea is becoming increasingly present. This is due, on the one hand, to the progressive integration of ICTs into the teaching-learning processes and, on the other hand, to the impact of these changes in terms of defining what DC is and how to develop it in schools. For this reason, this work focuses on explaining the scientific reality of the term “netiquette” through a review of the literature in the main databases. This is an approach to finding out and understanding the state of research into labels in a universe marked by haters, cyberbullying, and fake news.

## 2. Method

This systematic review is based on the analysis of existing literature in the Scopus and Web of Science (WoS) databases of the term netiquette. Its implementation has been developed in accordance with the Preferred Reporting Items for Systematic Reviews (PRISMA) [78] in order to answer the following questions. The structure of other publications in impact journals [79,80,81] has also been taken into consideration in order to follow models of analysis validated by experts. In turn, data from the studies analyzed are included, such as the country of origin, the date of publication, the main objectives, the methodological design, the variables considered, the details of the samples, and their scientific contributions to the area of research.

RQ1 What is the state of scientific production regarding “netiquette”?

RQ2 Has an interest in “netiquette” increased since the emergence of social networks?

RQ3 What is the scientific relationship between “netiquette” and the educational field?

### 2.1. Search Strategy

During the month of December 2019, a strategy was developed based on the search for articles that include the term “netiquette” in the title and that are part of two relevant scientific databases. Given the nature of this research, this restrictive criterion was chosen because otherwise the inclusion of articles that were not scientifically relevant to the research could be favored. In this sense, both terms were included in the Scopus and WOS search engines (WOS, BCI, BIOSIS, CCC, DIIDW, KJD, MEDLINE, RSCI, SCIELO), two scientific databases commonly used by experts and researchers and from which both JCR and SJR draw their information [82]. A single search criterion was established, the title of the article having to incorporate one of the two terms. This initial search yielded 53 manuscripts, although the final sample consisted of 18 references.

### 2.2. Inclosure Criteria

**Figure 1:**
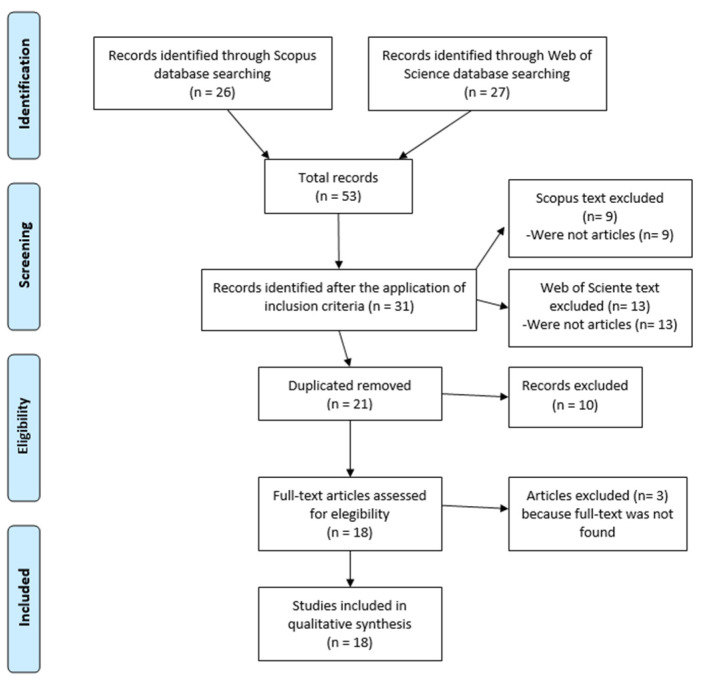
Flow diagram of PRISMA Systematic Review about “netiquette.”

The channeling of the results to the final sample was carried out on the basis of the PRISMA protocol [78] for carrying out systematic reviews. The main objective was to analyze those articles that really focused on “netiquette,” and so it was established as a search criterion that this term should appear in the title of the articles to be analyzed later. Afterward, those results that were not articles were eliminated, both in WOS (n = 13) and in the SCOPUS database (n = 9). Of the 31 resulting articles, having searched two databases, those that were duplicated and were part of both were eliminated (n = 10). Once they were deleted, the information available on the remaining 21 articles was analyzed to check their eligibility, and they were read in full in cases of doubt about their subject matter. Finally, those whose complete text could not be found on the Internet were eliminated (n = 3), leaving the final sample reduced to 18 articles (Figure 1). Articles included in the title “netiquette” or “netiquettes,” not repeated in the databases and with access to the full text.

## 3. Results

All the articles that have been considered for analysis were presented in English (n = 18). A consistent figure considering that more than half (n = 12) have been published from the UK (n = 4) or the US (n = 7). The time span between the oldest and most recent article is 23 years, covering 1995 and 2018. The focus of the articles can be grouped into two main blocks, empirical studies (n = 9) and theoretical approaches (n = 9), as shown in Table A1 and Table A2 respectively. The methodological disparity is clearly noticeable in the quantitative articles, with cases of quantitative (n = 4), mixed (n = 2), and qualitative (n = 1) approaches. (Appendixe A and Appendixe B).

### 3.1. Country

More than half of the articles studied were of Anglo-Saxon origin, specifically from the United Kingdom [61,64,70,76] and the United States [59,60,65,66,67,68,69,71]. Both cases stand out as they are the only countries that are repeated in terms of place of publication. The remaining (n = 6) come from European countries, such as Germany [78], Denmark [62] and Belgium [72]; from Asia, South Korea [54] and Jordan [74]; and from the American continent, Mexico [75]. Article [66] should be defined as having double authorship, from the United States and Canada. It should be noted that the United Kingdom [61,70] and the United States [59,60,65,66,67,68,69] are the only two countries that contribute articles of a theoretical nature, while those with an empirical focus are more spread out around the world.

### 3.2. Date

There is a disparity in the date of publication of articles. With respect to the empirical ones, the oldest is from 2007 [72] and the most recent from 2018 [77], with only repetitions in 2017 [62,74]. In fact, all the articles are from the last decade [54,62,64,71,74,75,76,77] except the one from 2007. On the other hand, those theoretical approaches cover the period from 1995 [61] to 2018 [67] and there are also repetitions in 2011 [59,68]. By decade of publication, production stands out from 2000 to 2010 [65,66,69,70], from 2010 to 2020 [59,67,68], and from 1990 to 2000 [60,61]. Of the total, only four articles [62,67,74,77] have been published during the last five years.

### 3.3. Aims

On the one hand, the objectives of the empirical articles can be differentiated into those more linked to netiquette in educational contexts [54,71,72,74,75,77] and those oriented to more general personal or work environments [62,64,76]. In the majority of articles [54,62,64,72,74,75,76,77] the objective is based on knowing habits associated with the label on the net, in some cases, the objective is purely methodological [71]. As for the theoretical articles, the distinction is less clear. Up to 5 [59,65,66,67,68] focus on exposing or analyzing guidelines related to the correct use of electronic mail and two [60,61] provide more general guidelines for the Internet in its complexity. Only three are developed for specific contexts: hospital workers [68,69] and librarians [70].

### 3.4. Methodological Design

Two clear methodological designs can be distinguished: empirical articles [54,62,64,71,72,74,75,76,77] and theoretical articles [59,60,61,65,66,67,68,69,70]. From the first case, there is a new differentiation: quantitative supported by ad-hoc questionnaires [54,64,74,77], mixed approaches [62,72], and only qualitative [75]. One of the articles is purely methodological [71], so its scientific contribution is different from the rest. In the case of theoretical studies, they can be divided into purely theoretical [59,65,66,67,68,69,70] and literature reviews [60,61].

### 3.5. Main Variables

The variables found in the articles analyzed are very diverse. The quantitative variables explore online time and its possible relationship with cyber-bullying [54] or peer-to-peer tagging [64], university students’ knowledge of it [74], or its direct application through interaction with faculty [77]. In the case of those based on a mixed methodology [62,72], they are based on category analysis (attitude, motivations, unsubstantiated statements, etc.,) and are interspersed with other numerical quantitative variables (questions, number of visits to the forum, number of times they read what is published in the forum, etc.,). The qualitative article [75], with a socio-historical perspective, is based on categories such as “moral practice,” “communities of practice,” and “netiquette.”

### 3.6. Sample Details

The samples in half of the articles analyzed [54,62,64,71,72,74,75,76,77] are very varied. They range from small groups of 34 secondary school students [75] to 992 couples [64] or 2849 students and teachers [77]. The educational context of the samples is relevant, as more than half [54,71,72,74,75,77] of the articles are composed of students or graduates. There are also undefined figures when exposing themselves based on groups [76] and samples where the only requirement was to have a Facebook account [62] or to have a partner [64].

### 3.7. Measurement

The instruments used in the articles analyzed cover quantitative [54,64,74,77], mixed [62,72], and qualitative [75,76] perspectives. In this sense, the quantitative instruments have been based on the development of questionnaires designed ad-hoc [54,64,74,77], the mixed ones have been questionnaires and subsequent coding, and the qualitative ones have employed interviewing and discourse analysis individually or through focus groups. The theoretical articles have not used instruments in their development.

## 4. Discussion

The last two decades have shown the capacity for technological development and the human ability to incorporate it into daily routines [9,10,11,31,32,35]. In the case of the Internet, its birth and evolution have meant a before and after in humanity [12,13,33,34], and has altered the way people communicate and collaborate [1,18]. Having and using a smartphone [21], even becoming addicted [51], or spending time on social networks [2] are new patterns of behavior in a society where digital skills are becoming essential [26,27,29,36,38]. So much so that in the educational field the relevance of the so-called DC [39,40,41,42,43,50] is increasing. In short, it is clear that these years have seen the birth of a new question that goes beyond ethics: how to behave on the Internet [20,28,37,38,60,61].

The analysis of the articles compiled through Scopus and Web Of Science, 18 of which finally passed the inclusion criteria set out through the PRISMA analysis process [54,59,60,61,62,64,65,66,67,68,69,70,71,72,74,75,76,77], leads to the following inferences. Despite the fact that the included literature covers a significant period of time, with a margin of several decades [60,61,62,74,77], the state of the search remains exploratory. There is a disparity between theoretical and empirical approaches, which accentuates the lack of a clear line of research. E-mail [59,65,66,67,68] and its network label are the main focus of theoretical articles, while in the case of empirical ones the characteristics of the samples are usually linked to educational [54,71,72,74,75,77] or training contexts.

In relation to the instruments indicated in the literature analyzed, the disparity in the methodologies and tools used stands out. The quantitative researchers base their analysis on ad-hoc questionnaires [54,64,74,77] whose scientific criteria are not clear, so it is complex to affirm their validity and that they are reliable. At the same time, the sample sizes are disparate, with figures that are either not very representative [62,75] or fairly representative [54,64,77]. On the other hand, methodologies supported by open questions, coded analysis of discussion groups, or field diaries have also been found. On no occasion are the objectives of two or more articles repeated or similar, each of the articles analyzed is supported by unique theoretical frameworks and instruments.

The results presented by the articles researched can be grouped into two aspects. Theoretically, the relevance of the correct use of electronic mail in the digital world is revealed through the presentation of guidelines and guides [59,65,66,67,68]. On the other hand, digital trends are shown, such as cyberbullying [54], mourning, and commemoration practices on Facebook [62], and the impact on couple relationships [64]. In the educational framework, there are complementary ideas such as the lack of knowledge of netiquette on the part of university students [74] and the improvement in the quality of discussion in forums when guidelines of this type are provided previously [72].

## 5. Conclusions

Considering the results found in this work, it is consistent to conclude that netiquette is a field of study that is in its initial phase. The limited production in this line of research is very significant, especially in view of the existence of theoretical articles from more than two decades ago. It is complex to consider that there is a real interest in research in this area. Defining an ethic for a context that changes almost daily is complex, however, it is necessary to understand it if we want to improve the society. The DC [39] includes netiquette as a training demand, both from students and teachers, and it is understood in reality that it includes digital economic sectors, the rise of cyberbullying [54], or the establishment of nomophobia [51].

Different considerations can be made with regard to the starting hypotheses. Scientific production relating to netiquette is still at an early stage, without a defined theoretical basis despite being a term that has existed since before the 21st century. The birth of social networks has indeed increased the interest in netiquette, at least in terms of new habits and specific ethical factors. The works published in the past decade take into account the existence of these new media, a vision that is coherent with how they have become internalized in the routine of billions of people. The selection of students in training, whether current or recent, is a scientific criterion that reinforces the link between education and netiquette. Digital preparation is a fundamental pillar in personal, social, and professional terms. It is therefore inevitable to associate both areas in the present without thinking about the future, something that is set out in the current conception of the DC.

In relation to the limitations of the present study, existing in the studies based on the systematic review, there is a risk of having lost information because of the strategy of selection of the descriptor. Introducing the term netiquette, and its plural, as the only search elements were established in view of its presence in educational and legislative frameworks. Some of the lines of research in this area that are proposed are the creation of new instruments to find out the level of preparation of students, teachers in training, or teachers.

In conclusion, this study presents a number of theoretical and practical implications. The implications in the educational field, after having carried out the analysis, imply the need to revise the digital preparation of all the agents that form part of this field. The theoretical and practical synthesis set out in this work may mean a new scientific stage of an essential issue for the 21st century. Specifically, to cite more specific examples, it can lead to the beginning of a realistic consideration of digital needs, demands, and capacities in everyday tools such as e-mail, social networks, and even others close to home. For this reason, this study not only offers a new line of work to researchers or experts from the scientific community but can also have repercussions for anyone in the world with access to digital devices, with a special interest in the educational context.

## Appendix A

**Table A1:** Empirical studies.

Ref.	Country	Date	Aim (s)	Methodology	Sample Details	Main Variables	Measurement	Main Findings	Implications
[54]	South Korea	2014	To study the relationship between levels of online activity and cyber-bullying behavior	Correlational. Random sampling.	1200 teenagers	Bullying. Cyberbullying. Netiquette. Time online. Type of activities. Use of social networks. Communication with parents.	Face-to-face survey	Frequent users of the Internet and social networks are more likely to participate, become victims and witness cyber-bullying.	It is necessary to take preventive measures with teenagers to avoid cyberbullying.
[62]	Denmark	2017	To analyze the rules underlying online mourning and commemoration practices on Facebook	Mixed. Qualitative, quantitative.	166 Danish Facebook users	Attitude. Caring for the deceased. Caring for the bereaved. Taking care of friends. Legitimate practices. Objectionable practices. Mourning. Remembrance. Need for support. Questionable motives. Privacy. Publicity.	Ad-hoc questionnaire and coding with NVivo10	Findings counter popular perceptions of Facebook as a desired online grief platform.	Despite not being the preferred medium, social media are a common means of communication with deep thematic.
[64]	United Kingdom	2010	To examine whether married couples have similar ideas about network etiquette.	Quantitative.	992 married couples	Netiquette. Use of the Internet. Specific activities. Supervision.	Adaptation of the eHarmonny survey.	A netiquette is developed and negotiated consciously or unconsciously in intimate relationships.	
[71]	United States	2012	To present a methodological proposal based on the incorporation of laptops in the classroom.	Methodological article	356 students	Use of laptop computer. Qualifications. Distraction	Ad-hoc survey	The majority of the students surveyed consider the accepted methodological policy to be positive. The proposal is based on placing the students who use the laptops in the first rows and there are point sanctions if there is a misuse or invented warning.	The incorporation of ICTs in the classroom can be functional and educational, but it is necessary to establish guidelines and consensus for students to understand in this way.
[72]	Belgium	2007	To investigate whether the type of guideline provided has an effect on the quality of asynchronous group discussion or on participant assessment in the context of a medical course.	Experimental. Content analysis.	112 graduate students in biomedical sciences.	Number of visits to the discussion forum. Number of times they read what has been published in the forum. Questions. Arguments. Unsubstantiated statements.	Discussion groups.	The group that received educational guidelines and advice on network etiquette had a higher quality of discussion and evaluation by the participants. There was no impact on the group that only received guidelines on network etiquette.	The more information students are provided with, the better they will understand digital formality.
[74]	Jordan	2017	Study the presence of netiquette practices among university students.	Descriptive research.	245 university students (125 classroom teachers and 120 special education teachers)	Gender. Specialization. Level of study.	Ad-hoc questionnaire. Likert type.	University students have a consensus on the general rules of netiquette, limited knowledge of them and different levels of implementation, Limited practice of netiquettes related to critical thinking skills.	There is a consensus on rules on the Internet, but it’s development and critical capacity needs to be further developed.
[75]	Mexico	2015	To offer a panorama based on how moral practices develop ah now the rules of netiquette are applied in communities formed by secondary school students in their practices of virtual interaction.	Qualitative with a socio-historical perspective. Ethnography.	34 students secondary education.	Categories. Moral practice. Communities of practice. Netiquette.	Open-ended questionnaire, field journal and an unstructured group interview.	Students consider morality and attachment to the family to be positive ideals that can be achieved, but exercise free behavior in virtual interactions.	There are discrepancies between knowing and doing on the Internet. Attention should be paid to ensuring that students apply what they know.
[76]	England	2011	To examine the concept of agreement, how and why it is reached in an online interprofessional group.	Qualitative. Discourse analysis.	Ten interprofessional discussion group	Agreement. Disagreement. Online communication.	Discourse analysis.	Students tend to agree with each other’s comments rather than provoke disagreement.	In professional contexts, consensus is quickly reached. This is far from the reality in media such as social networks.
[77]	Germany	2018	To examine the netiquette for Facebook contacts between students and their teachers.	Multiple closed answers.	2849 participants (2550 students and 299 teachers)	Development of SL-Contacts. Netiquette and majority.	Ad-hoc questionnaire.	Most participants indicated that Facebook should be used only for private matters. The appropriateness of social networking contact between students and teachers depends on individual cases.	The use of social networks for educational purposes is not valued. It is recommended to focus on digital tools that are clearly intended for educational purposes.

## Appendix B

**Table A2:** Theoretical studies.

Reference	Country	Date	Aim (s)	Methodology	Main Findings
[59]	United States	2011	Define the concept of a networked label and include guidelines to ensure that electronic communication takes place in an appropriate and polite manner.	Theoretical article	Different guidelines are set out to encourage written communication via e-mail. Some of them are: to use grammar and punctuation correctly, to avoid excessive use of abbreviations and acronyms, to use emoticons only, not to use the “high priority” option, to use a signature with personal contact information, to use spaces to avoid long messages, to avoid always using capital letters, to enter correctly and include a well-defined subject, to avoid sending sensitive information by e-mail, to avoid writing during other interactions.
[60]	United States	1997	Attempt to collect and develop standard label guidelines in the context of a global Internet.	Literature review	The term netiquette has been described for e-mails and Internet use. A collection of authors is made on patterns of behavior on the Internet, specific suggestions, rules of network etiquette for advertising, control of undesirable network etiquette, the influence of Internet services, employees, and governments.
[61]	United Kingdom	1995	Identify, present and digest some of the main patterns of netiquette	Literature review	The article presents different guidelines contained in different publications based on a total of 20: focus on objective, short and concise messages, edit your quotes, write grammatically correct, consider expressive typography, sign your messages, think where you want to go, mistakes can last forever, know the acronyms, don’t talk to a computer, don’t write in capital letters, try another kind of humor, think before you write, respect intellectual rights, be polite to newcomers, solve the necessary in private, be an ethical user, don’t damage the network, be proud of what you post, there is no rule 20.
[65]	United States	2004	Present guidelines to alleviate problems in communication through email or phone calls.	Theoretical article	It presents 15 guidelines for personal writing of emails (always include a subject in the message, do not use capital letters, use appropriate language, use emoticons,...) and 11 guidelines for sending emails in distribution lists or groups (publish only what is relevant to the group, ask questions or comments without losing the focus of discussion, give feedback when you can, ask permission before sending large proposals to the organizer or moderator).
[66]	United States/Canada	2002	Presenting some guidelines for e-mail etiquette.	Theoretical article	Different issues are presented in relation to e-mail: characteristics (backup, password protection, network and control systems, the threat of viruses, legal implications), risks (visual importance, avoid too much content, include emoticons, be careful with abbreviations), other risks (do not send negative information without notice, indicate response or delivery deadlines, use CC or Bcc) and practices to follow (be brief and concise, include a suitable subject, include a signature at the end, consider quoting a message or writing a new one, don’t send mass mailings, separate your personal mail from the professional one, keep your distribution lists updated, don’t open a mail if you don’t trust the source, don’t forget to say hello and goodbye.
[67]	United States	2018	To provide the tools to avoid problems in electronic communication through email.	Theoretical article	It provides different guidelines regarding network behavior (basic rules such as using a professional email in a professional context, including subject, being concise, responding quickly, or forwarding emails only with permission). Also what not to do (offensive language, using capital letters, or avoiding emoticons in professional contexts), the negative impact (virtual empathy). It includes netiquette guidelines for an online learning environment, case studies, “the golden rules of netiquette” and the importance of positive communication.
[68]	United States	2011	Provide a total of 50 rules for network etiquette for e-mail. Intended for employees in medical practice.	Theoretical article	It turns out to be a compilation of different guidelines, what to do and what not to do, regarding e-mail in the professional medical context. Some examples are: be concise, avoid long sentences, use templates, use a contact signature, protect the privacy of others, turn off the automatic reply, respect confidentiality, do not abuse the “high priority” option, do not write everything in capital letters, do not remember messages, do not ask for too much, do not use abbreviations, do not expect privacy when using a work email, etc.
[69]	United States	2000	Guidelines for the use of appropriate distribution lists by nurses in their professional context	Theoretical article	Different ethical and practical issues for the use of distribution lists in the context of nursing are presented. Respect the ethical code (maintain privacy, provide information and sources for ethical decisions, incorporate legislative framework), avoid unethical messages (ask questions), consider Internet privacy, practical suggestions (do not leave your email account open and go away, sign your message, do not incorporate advertising, do not publish institutional messages without permission, do not write disrespectful or insensitive messages).
[70]	United Kingdom	2002	Expose the importance of confidentiality among librarians and users in the face of the attraction of new technologies.	Theoretical article	Taking as a reference to a study by Loughborough University, which exposed the confidence of users and the poor preparation of librarians, a series of ethical reflections are raised. The development of specific users in libraries, individuality and privacy, access to the Internet and the individual, punishment, harassment, handling information, and making good policies.
